# Optimizing Secondary Electrospray Ionization High-Resolution Mass Spectrometry (SESI-HRMS) for the Analysis of Volatile Fatty Acids from Gut Microbiome

**DOI:** 10.3390/metabo10090351

**Published:** 2020-08-28

**Authors:** Jisun H. J. Lee, Jiangjiang Zhu

**Affiliations:** 1 Department of Human Sciences, The Ohio State University, Campbell Hall, 1787 Neil Avenue, Columbus, OH 43210, USA; lee.9639@osu.edu; 2 James Comprehensive Cancer Center, The Ohio State University, Wiseman Hall, 400 W 12th Ave, Columbus, OH 43210, USA

**Keywords:** secondary electrospray ionization (SESI), volatile fatty acids (VFAs), gut microbial metabolism, antibiotics

## Abstract

Gut microbiota plays essential roles in maintaining gut homeostasis. The composition of gut microbes and their metabolites are altered in response to diet and remedial agents such as antibiotics. However, little is known about the effect of antibiotics on the gut microbiota and their volatile metabolites. In this study, we evaluated the impact of a moderate level of ampicillin treatment on volatile fatty acids (VFAs) of gut microbial cultures using an optimized real-time secondary electrospray ionization coupled with high-resolution mass spectrometry (SESI-HRMS). To evaluate the ionization efficiency, different types of electrospray solvents and concentrations of formic acid as an additive (0.01, 0.05, and 0.1%, *v*/*v*) were tested using VFAs standard mixture (C_2_–C_7_). As a result, the maximum SESI-HRMS signals of all studied m/z values were observed from water with 0.01% formic acid than those from the aqueous methanolic solutions. Optimal temperatures of sample inlet and ion chamber were set at 130 °C and 85 °C, respectively. SESI spray pressure at 0.5 bar generated the maximum intensity than other tested values. The optimized SESI-HRMS was then used for the analysis of VFAs in gut microbial cultures. We detected that the significantly elevated C_4_ and C_7_ VFAs in the headspace of gut microbial cultures six hours after ampicillin treatment (1 mg/L). In conclusion, our results suggested that the optimized SESI-HRMS method can be suitable for the analysis of VFAs from gut microbes in a rapid, sensitive, and non-invasive manner.

## 1. Introduction

Over the past two decades, the mass spectrometry-based methodologies such as proton-transfer-reaction mass spectrometry (PTR-MS) and selected ion flow tube mass spectrometry (SIFT-MS), in addition to the gold-standard gas chromatography-mass spectrometry (GC-MS), have been used for the detection and analysis of gas-phase volatile organic compounds (VOCs) [1,2,3,4]. In recent years, secondary electrospray ionization mass spectrometry (SESI-MS) has also emerged for the studies of gas-phase VOCs from yeast, plant, bacteria, and breath [5,6,7,8]. SESI-MS is a real-time, non-invasive, and sensitive technique with a reported limit of detection at as low as 0.2 parts per trillion by volume (ppt) [9]. In addition, SESI coupling to high-resolution mass spectrometry (HRMS) has been shown to enhance the capability to capture as many VOC metabolites as possible in real-time mass spectrometric analysis [10].

SESI was invented as a variant of the electrospray ionization source (ESI) that can be specifically utilized for gaseous analytes [11,12]. It is an ambient electrospray ionization type that can produce charging agents from evaporating nano-droplets/ions to charge neutral vapor molecules in the gas phase [13,14]. Many factors, including the yield of the charged ions, charge transfer rate, and airflow field condition, can influence the efficiency of ionization of SESI [15]. While efforts have been made to understand the exact ionization mechanism of SESI, the impact of some major SESI parameters on its signal intensities and ionization efficiency remains to be fully defined [14].

The gut microbiome plays an important role in maintaining gut homeostasis, and gut microbial VOCs are considered as potential biomarkers in defining gut health [16]. Previous studies demonstrated that the dysbiosis of the gut microbiota is linked to metabolic disease through changes to microbiota-derived metabolites, including volatile fatty acids (VFAs) via fermentation [17]. Microbial VFAs have beneficial effects in protecting intestinal epithelial barrier integrity and against intestinal inflammation [18]. In general, VFAs include a group of aliphatic monocarboxylic acids with different lengths of the chain containing from 2 to 7 carbon atoms, namely acetic (C_2_), propionic (C_3_), iso-butyric and butyric (C_4_), iso-valeric and valeric (C_5_), iso-caproic and hexanoic (C_6_), and heptanoic (C_7_) acids [19]. The majority of them are also known as short-chain fatty acids (SCFAs) according to the main structure (C_2_–C_6_). The gut microbial SCFAs are attributed to their attendant responses to the host lifestyle such as dietary composition and intervention [20]. Moreover, SCFAs have been associated with obesity and diabetes and their related dysbiosis of the gut microbiota [21]. Consequently, various clinical studies have demonstrated SCFAs as potential biomarkers for metabolic diseases [22,23,24]. It has been reported that the extrinsic factors, such as diet and antibiotics, perturbate the gut microbiome community and their metabolites, which may lead to dysbiosis [17,25]. The administration of antibiotics has been widely applied in the treatment and prevention of infectious diseases [26]. However, fewer studies have been conducted to evaluate the effect of antibiotics on the VFA profiles of the gut microbiota [26,27].

In this study, we performed a series of experiments to optimize the SESI parameters, including the types of electrospray solution, temperatures of sample inlet and ion chamber, and SESI spray pressure, for the sensitive and specific detection of VFAs from chemical standards. In addition, the optimized method was used to evaluate the effect of a moderate concentration of ampicillin on VFAs’ profiles from the headspace of gut microbial cultures.

## 2. Results and Discussion

### 2.1. Optimizing SESI Conditions for the Best Performance of Gas-Phase VFA Analysis

#### 2.1.1. Identification of Six Representative m/z Values

**Scheme 1:**
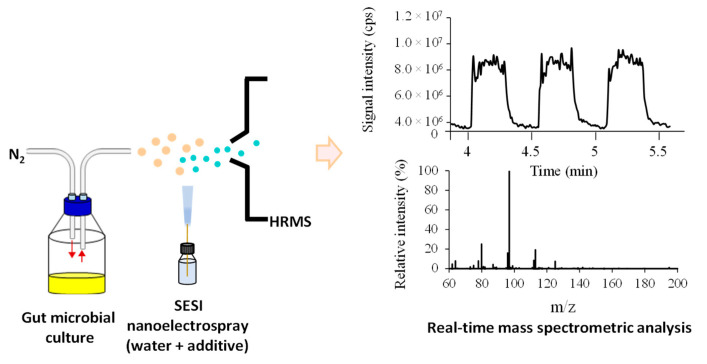
Schematic diagram of secondary electrospray ionization mass spectrometry (SESI-HRMS) for the analysis of gas-phase volatile organic compounds (VOCs) in the headspace of gut microbial culture.

The present study focused on evaluating the impact of different parameters on real-time SESI analysis so that the optimized conditions can eventually be used to analyze C_2_–C_7_ volatile organic acids in the headspace of gut microbial culture. The optimized parameters included different types of spray solvents, concentrations of formic acid, temperatures of sample inlet and ion chamber, and SESI spray pressure. A schematic diagram of SESI-HRMS analysis steps using gut microbial cultures is given in Scheme 1.

To optimize SESI parameters, 50 µmol/L of fresh standards of a volatile free acid mixture consisting of acetic acid (C_2_-VFA), propionic acid (C_3_-VFA), isobutyric acid/butyric acid (C_4_-VFA), isovaleric acid/valeric acid (C_5_-VFA), isocaproic acid/hexanoic acid (C_6_-VFA), and heptanoic acid (C_7_-VFA) were diluted in deionized water and used as the source of volatiles for SESI-HRMS detection. The headspace VFAs of the standard mixture were introduced into the SESI-HRMS system as the same way to detect microbial VFAs, as shown in Scheme 1. The identification of six representative m/z features, including 59.0138 (C_2_-VFA), 73.0296 (C_3_-VFA), 87.0453 (C_4_-VFA), 101.0609 (C_5_-VFA), 115.0765 (C_6_-VFA), and 129.0922 (C_7_-VFA) were confirmed based on the characteristics of mass accuracy (Table 1).

#### 2.1.2. Optimization of Essential SESI Parameters

**Figure 1:**
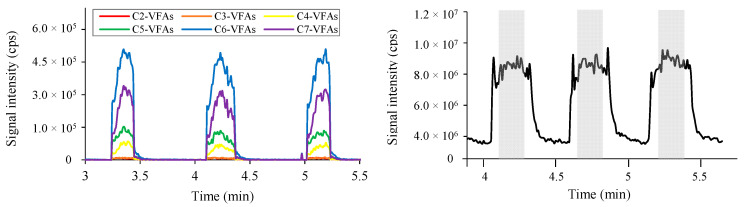
Online detection of three consecutive sampling process. (**A**) Time traces of the corresponding metabolites of C_2_–C_7_ volatile fatty acids (VFAs) in the headspace of standard solutions, (**B**) Each sampling remains for 15 s and the gray-colored highlighted range was selected for signal intensity analysis via manual peak integration.

The diluted VFA mixture was used to optimize a set of essential SESI parameters. The initial conditions of SESI parameters (water spray solution supplemented with 0.01% formic acid (*v*/*v*), sample inlet and ion chamber temperatures at 70 °C and 80 °C, respectively; and a SESI spray pressure set at 1 bar) were optimized one at a time. Figure 1 presents the visualized typical workflow for gut microbial VOCs data obtained with SESI-HRMS for online analysis. Figure 1A depicts the total ion counts (TIC) with time tracts of the corresponding m/z features (C_2_‒C_7_ VFAs) over the three consecutive sampling processes for 15 s each. Figure 1B shows the standardized sampling process, which is similar to that used in previous studies [28]. The gray-colored parts indicate detection of time points of VFAs analysis and the areas have been integrated for semi-quantitative peak intensity analysis (Figure 1B).

##### Electrospray Solvents

**Figure 2:**
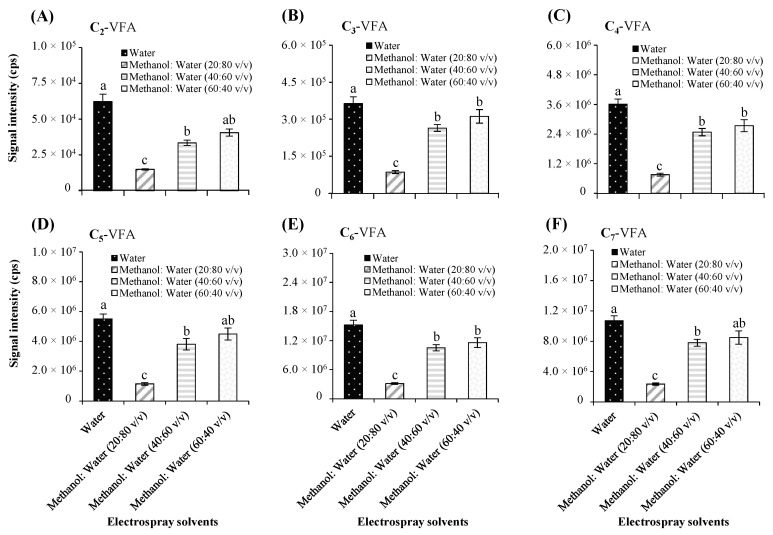
The effect of electrospray solutions on signal intensities of six targeted m/z features of detected C_2_–C_7_ VFAs, (**A**) C_2_ VFA, (**B**) C_3_ VFA, (**C**) C_4_ VFA, (**D**) C_5_ VFA, (**E**) C_6_ VFA, (**F**) C_7_ VFA, were tested using four types of electrospray ionization source (ESI) solutions. Three replicates were used to examine the effect of optimized and least optimized SESI conditions on signal intensities of volatile fatty acids (C_2_‒C_7_) in the headspace of diluted VFA standard mix by performing the Tukey’s post-hoc test, and the results were expressed using bar graphs with mean ± standard error. Different letters indicate a statistically significant difference (*p* < 0.05).

The selection of the electrospray solvent which is implicated in the chargeability of the analyte is crucial for ionization in the electrospray process in SESI-HRMS analysis [14,29]. Initially, water and different methanol/water solutions, including 20:80 (*v*/*v*), 40:60 (*v*/*v*), and 60:40 (*v*/*v*) with 0.1% formic acid (*v*/*v*) were prepared to examine the impact of spray solution choice to mass spec peak intensities of VFAs (Figure 2). The significant differences in the signal intensity of targeted C_2_–C_7_ VFAs were observed based on the different types of tested spray solutions. In Figure 2, pure water with 0.1% formic acid shows the highest signal intensity than aqueous methanolic solutions in this study. Notably, substantially lower levels of signal intensities of all studied C_2_–C_7_ VFAs were observed in the methanol/water mix (20:80, *v*/*v*) condition, followed by the methanol/water (40:60 and 60:40, *v*/*v*). Therefore, the water with 0.1% formic acid (*v*/*v*) condition was selected for optimizing further parameters. It has been reported that electrosprays of pure solvents can efficiently ionize gas-phase analytes, and several types of electrospray solutions such as water and methanol have been used for various studies [30,31,32]. An earlier study by Sinues et al. [33] examined the efficiency of nano-electrospray ionization using three types of spray solvents, including water, water/methanol (1/1), and methanol, to measure amine-containing compounds such as dimethylamine, ethylamine, and trimethylamine. Similar to our results, the highest signal sensitivity can be observed from water sprays, and the lowest ones were detected with methanol solution [33].

##### Concentration of Formic Acid as an Additive in Electrospray Solvent

**Figure 3:**
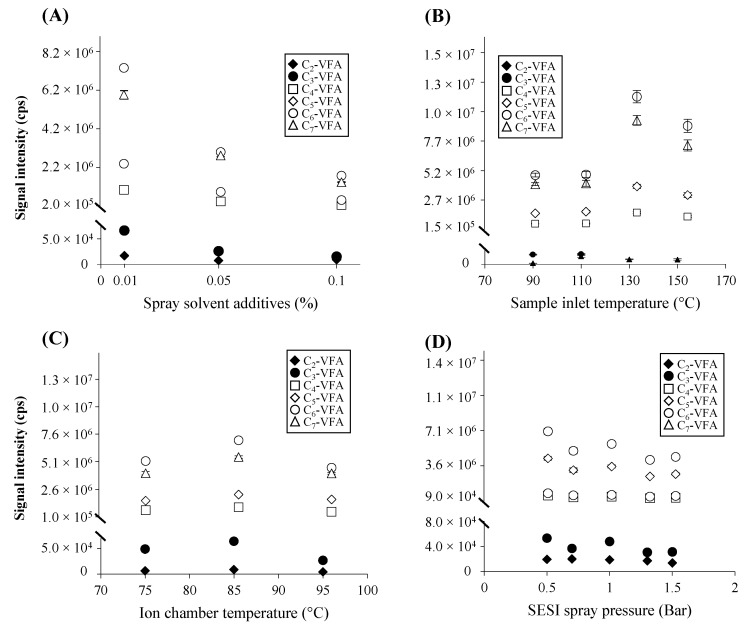
Optimization of SESI parameters. (**A**) Comparison of different concentrations of formic acid (0.01, 0.05, and 0.1%) prepared in water as spray solvents, (**B**) Comparison of sample inlet temperatures (90, 110, 130, and 150 °C), (**C**) Comparison of ion chamber temperatures (75, 85, and 95 °C), (**D**) SESI spray pressures (0.5, 0.7, 1, 1.3, and 1.5 bar). Three replicates were used to examine the effect of optimized and least optimized SESI conditions on signal intensities of volatile fatty acids (C_2_‒C_7_) in the headspace of diluted VFA standard mix by performing the Tukey’s post-hoc test, and the results were expressed using bar graphs with mean ± standard error. Different letters indicate a statistically significant difference (*p* < 0.05).

To determine the influence of formic acid, three different formic acid concentrations, 0.01%, 0.05%, and 0.1%, were analyzed to evaluate the signal intensity of representative six m/z features (Figure 3A). The highest signal intensities of all studied VFAs were found from water spray solvent supplemented with 0.01% formic acid (*v*/*v*) than the values detected from spray solvent with 0.05% and 0.01% formic acid (*v*/*v*). Therefore, 0.01% and 0.1% formic acid concentrations in water were chosen as the optimized and least optimized conditions for VFA analysis, respectively, in the later part of this study. Previously, several types of additives, including formic acid, acetic acid, and ammonium formate, have been used to facilitate the interactions between analytes and electrospray charged droplets to enhance the sensitivity of SESI analysis [30,32,34]. It has been proposed that gas-phase analytes could be charged by contacting either electrospray droplets or protons that are generated after the droplets have evaporated [33]. However, the exact way of ion production during the SESI ionization process has not been fully understood [14]. Our result may imply that the lowest level of formic acid (0.01%) contributes to the production of higher signal intensity by enabling gas-phase proton affinity of the target analytes or the production of stable negative ions for VFAs analysis in this study [35].

##### Sample Inlet and Ionization Chamber Temperature

The sampling line and ionization chamber sections of our SESI are silica-coated to minimize analyte adsorption onto the system walls. Therefore, optimizing the thermal conditions of both compartments is essential for maximizing operational efficiency. In this study, various thermal conditions such as 90 °C, 110 °C, 130 °C, and 150 °C were assessed to understand the impact of temperatures of the sampling line. Figure 3B shows that the maximum signal intensities of C_2_–C_7_-VFAs were observed by setting the sample inlet temperature at 130 °C, whereas the lowest signal abundances were achieved at 90 °C. Notably, the temperature-related signal intensity changes showed that gradually increased signal from 90 °C up to 130 °C, then decreased intensity was observed at 150 °C. The temperature of the sampling line is one of the influential factors which may affect the rate of analytic efficacy by minimizing adsorption of volatile metabolites onto the line and introducing them into a cylindrical SESI-chamber [30]. Different temperatures of the sample inlet (80‒160 °C) have been adopted in previous researches using SESI-MS [5,30,34,36,37]. Similarly, a recent study by Weber et al. analyzed breath VOC profiles of children with cystic fibrosis by setting a sampling line at 130 °C [8].

Subsequently, we examined the thermal influence of the ionization chamber on the six targeted m/z values of VFAs by setting three different temperature points, including 75, 85, and 95 °C (Figure 3C). Substantial thermal effects were detected on the signal intensity, and the optimal temperature was determined as 85 °C, at which the highest intensity of all studied m/z features was detected. The VFA peak intensities were then followed by detecting the standards at ionization temperature of 75 °C and 95 °C. It has been reported that the analytes in the gas phase are continuously introduced into the ion chamber by nitrogen carrier gas through the opening of the sampling line tube; the sampling line tube is then connected to the core of the SESI ion chamber. In the chamber, the analytes interact with the electrospray cloud and become positively or negatively charged as a result of ion-vapor reactions [33]. The temperature of the ion chamber may play a crucial role in enhancing the efficiency of sample analysis by avoiding the adsorption of sample vapors to the chamber surface [38]. It has also been reported that water dissociation constant *k*_w_ is temperature dependent [39], which suggests that the temperature differences in this test would also influence the abundance of protons from the SESI solution, and therefore impact the ionization process of SESI. In this study, the ionization chamber temperatures were heated to 75, 85, and 95 °C based on the literature reported range between 75 and 95 °C [30,32,34]. Interestingly, our result shows that the optimal temperature is 85 °C for C2–C7 VFAs analysis, and the decreased intensity has been observed at 95 °C. In addition, the boiling point of the specific electrospray solutions used for a particular study may need to be considered to screen the temperature point [30].

##### SESI Spray Pressure

Finally, five different points of SESI spray pressure (0.5, 0.7, 1.0, 1.3, and 1.5 bar) were set to drive the nano-electrospray solution through the silica capillary emitter (a 20-μm ID TaperTip (New Objective, Woburn, MA, USA) (Figure 3D). The different effect was observed based on the tested m/z features, and the significantly higher signal intensities of C_4_-C_7_-VFA were found at 0.5 bar, whereas no considerably different intensities were observed in C_3_- and C_2_-VFA between 0.5 and 1 bar, and 1–1.3 bar, respectively. Therefore, 0.5 bar was considered as the optimized value for producing the maximum intensity of all six m/z values, and 1.3 bar was selected as the least optimized condition. SESI pressure plays an important role in enabling a constant spray solvent delivery to the ion chamber to ionize analytes and generate robust results [38]. Our results may imply that a higher rate of SESI spray pressure dilutes sample vapors, which potentially lead to lower signal intensities.

In summary, the optimized SESI parameters to analyze of C_2_–C_7_-VFA in this study were determined as the following: 0.01% formic acid (*v*/*v*) in water as the preferred spray solution; sample inlet and ion chamber temperatures at 130 °C and 85 °C, respectively; SESI spray pressure of 0.5 bar. For further analysis, we analyzed the peak areas of studied m/z, which were obtained by the optimal SESI condition by comparing those produced from the least condition to show the significance of optimization of SESI parameters. The least conditions of each parameter were selected as the following: 0.1% formic acid (*v*/*v*) in water as the spray solution; sample inlet temperature of 90 °C; ion chamber temperature of 95 °C; and SESI spray pressure of 1.3 bar. Herein, further analyses were conducted by applying the optimal conditions of SESI parameters to detect VFAs, and comparing the results to the least optimized condition in headspace analysis of gut microbial cultures.

#### 2.1.3. Calibration Curve of Representative VFAs

**Figure 4:**
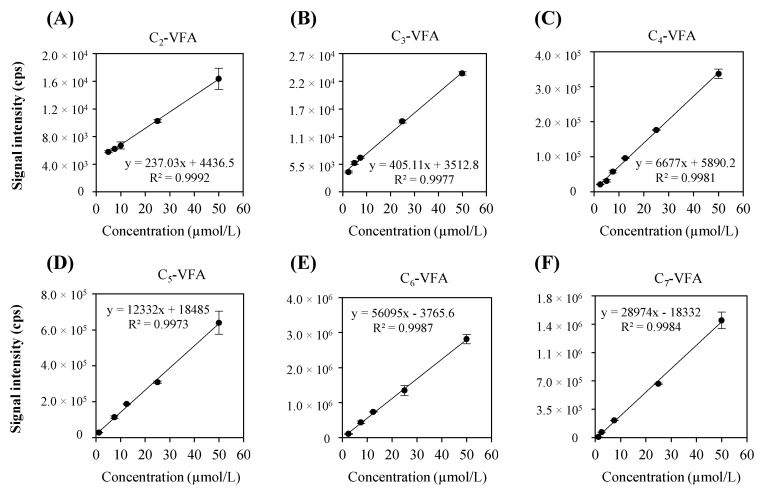
Calibration curves of C_2_–C_7_ volatile fatty acids (VFAs) using serial diluted standard mixture solution. (**A**) C_2_ VFA, (**B**) C_3_ VFA, (**C**) C_4_ VFA, (**D**) C_5_ VFA, (**E**) C_6_ VFA, (**F**) C_7_ VFA. Three replicates were used to examine the effect of optimized and least optimized SESI conditions on signal intensities of volatile fatty acids (C_2_‒C_7_) in the headspace of diluted VFA standard mix by performing the Tukey’s post-hoc test, and the results were expressed using bar graphs with mean ± standard error. Different letters indicate a statistically significant difference (*p* < 0.05).

To obtain calibration curves of six representative m/z features, C_2_–C_7_-VFAs, serially diluted solutions of fresh standards of volatile free acid mixture were prepared in deionized water. In Figure 4A, a good linear relationship between the total signal and the concentration of C_2_-VFA (5, 7.5, 10, 25, and 50 µmol/L) was observed with R^2^ = 0.9992. Similarly, decent linearities from C_3_-VFA (2.5, 5, 7.5, 25, and 50 µmol/L, R^2^ = 0.9977), C_4_-VFA (2.5, 5, 7.5, 25, and 50 µmol/L, R^2^ = 0.9981), C_5_-VFA (1.25, 7.5, 12.5, 25, and 50 µmol/L, R^2^ = 0.9973), C_6_-VFA (2.5, 7.5, 12.5, 25, and 50 µmol/L, R^2^ = 0.9987), and C_7_-VFA(1.25, 2.5, 7.5, 25, and 50 µmol/L, R^2^ = 0.9984) were achieved and demonstrated in Figure 4B–F, correspondingly.

### 2.2. SESI-HRMS Based Evaluation of Gas-Phase VFAs from Gut Microbial Culture

**Figure 5:**
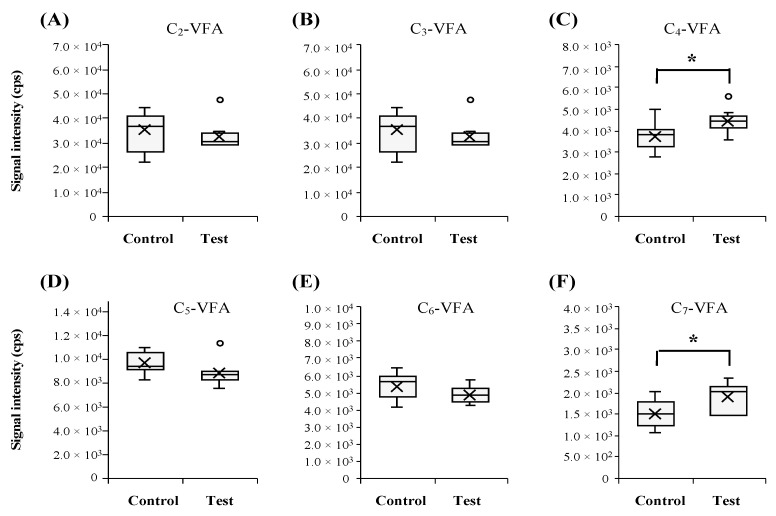
Comparative analysis between control and a moderate ampicillin-treated test group (1 mg/L). The signal intensities of six C_2_–C_7_ volatile fatty acids (VFAs) were evaluated six hours after antibiotic-treatment under the anaerobic condition at 37 °C. (**A**) C_2_ VFA, (**B**) C_3_ VFA, (**C**) C_4_ VFA, (**D**) C_5_ VFA, (**E**) C_6_ VFA, (**F**) C_7_ VFA. Three biological replicates were used to examine the effect of optimized and least optimized SESI conditions on signal intensities of volatile fatty acids (C_2_‒C_7_) in the headspace of gut microbial cultures by performing the Tukey’s post-hoc test, and the results were expressed using bar graphs with mean ± standard error. *, X, and O indicate statistically significant difference (*p* < 0.05), mean values, and outlier, respectively.

After establishing the optimized VFA detection method using our SESI-HRMS approach, our next step is to test if the method is sensitive in detecting the changes of VOC metabolites from the headspace of gut microbial cultures. In this study, antibiotic ampicillin (1 mg/L) was used as an example of treatment to the gut microbial cultures to mimic potential clinical conditions that antibiotics were prescribed for infection treatment. The volatile metabolites of the headspace of control (non-antibiotic treated) and test group (ampicillin-treated) were examined by SESI-HRMS. The OD values of control and ampicillin-treated groups were measured before and after treatments to investigate the effect of ampicillin treatment in microbial cultures, as well as to normalize signal intensities. As a result, there was no difference in the OD value of the control group between before (1.04 ± 0.04) and after (1.04 ± 0.02) treatments. However, the significantly decreased OD value (1.01 ± 0.02) was observed in the test group after 6 h post ampicillin treatment than the initial value (1.15 ± 0.05) before treatment (*p* < 0.05). The signal intensities of six representative volatile fatty acids (C2–C7 VFAs) in headspaces of microbial cultures were analyzed to examine the influence of ampicillin treatment on the VFAs (Figure 5). Notably, the intensity of C_4_ and C_7_ VFAs (Figure 5C,F) was significantly enhanced in ampicillin-treated groups compared to the control group (*p* < 0.05). Earlier studies reported that antibiotics treatment may reduce the levels of gut microbial VFAs according to the reduction in gut microbiota diversity [40]. Similarly, we also observed relatively decreased levels of C_2_, C_3_, C_5_, and C_6_ VFAs of test groups (Figure 5A,B,D,E), which may be led by the considerably reduced OD values (*p* < 0.05) after anaerobic incubation of antibiotic-treated cultures for six hours. Among the studied VFAs, C_4_ VFA such as butyric acid has received an increased attention because of its beneficial effects on the gut as a key energy source for colonic epithelial cells [26]. It was also reported to exert various intestinal activities that are involved in modulating intestinal epithelial defense and improving inflammation and intestinal motility [41,42,43]. In addition, previous studies have underlined the potential of butyric acid as a diagnostic biomarker associated with colorectal cancer based on the finding that the reduced bacteria species, such as *Ruminococcus* spp. and *Pseudobutyrivibrio ruminis*, produced butyrate [44,45]. Our results imply that a moderate dose of ampicillin treatment may alter microbial VFAs in a metabolite specific manner. It has been reported that the impact of antibiotics on the gut microbiome can be attributed to its antimicrobial spectrum and the antimicrobial activity in the colon depending on concentrations [46,47]. Previous studies examined the effect of several types of antibiotics on the perturbation of gut microbiota, which may lead to an alteration in colonic mucosal homeostasis and microbial metabolites [18,27,48,49]. Høverstad et al. reported that considerably changed SCFA profiles were observed according to the administration of ampicillin (500 mg four times daily) in the human clinical trial [46]. However, the consequences of short-term and long-term antibiotic use on the human microbiome with different types and dosages may need to be further analyzed and systematically summarized to understand the predictive values of SESI-HRMS’ results to the gut microbial metabolism and host health.

#### 2.2.1. Comparison of Optimized and the Least Optimized Parameters

**Figure 6:**
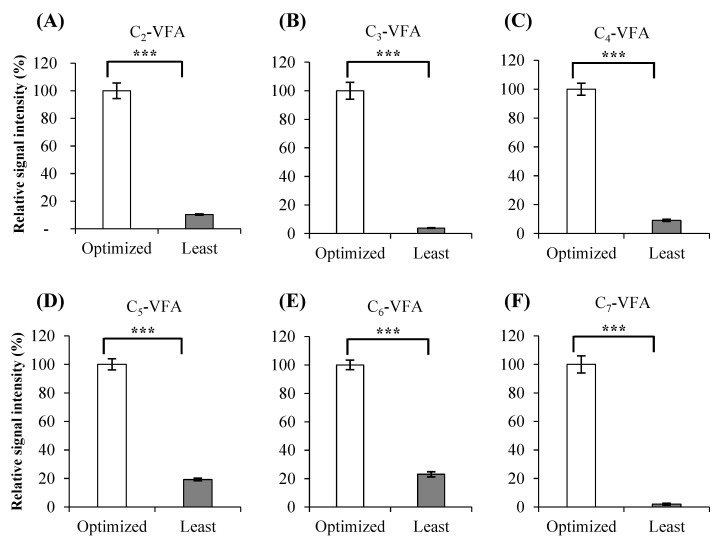
Relative signal analysis between the optimized and the least optimized SESI conditions according to the production of signal intensities. A significant difference (*p* < 0.001) was observed from all tested six C_2_–C_7_ volatile fatty acids (VFAs), (**A**) C_2_ VFA, (**B**) C_3_ VFA, (**C**) C_4_ VFA, (**D**) C_5_ VFA, (**E**) C_6_ VFA, (**F**) C_7_ VFA. Three biological replicates were used to examine the effect of optimized and least optimized SESI conditions on signal intensities of volatile fatty acids (C_2_‒C_7_) in the headspace of gut microbial cultures by performing the Student t-test, and the results were expressed using bar graphs with mean ± standard error. *** indicates statistically significant difference (*p* < 0.001).

To understand the essentiality of the optimization of SESI parameters, we compared the signal intensity achieved from the optimal condition to those levels from the least optimized condition, again using the ampicillin-treated gut microbial cultures. The combination of SESI parameters based on the least optimized signal intensities is the following: water as a spray solvent supplemented with 0.1% formic acid, 90 °C and 95 °C for sample inlet and ion chamber temperatures, respectively, and a spray pressure of 1.3 bar. Results in Figure 6 showed that the signal intensities of measured C2–C7 VFAs in comparison of the optimized condition versus the least optimized condition, and the results indicated that optimizing SESI conditions may be a prerequisite for a successful SESI-HRMS based application, as according to our observation, the optimal SESI condition outperform the least optimal conditions by a minimum of five-fold of signal increase (*p* < 0.001).

#### 2.2.2. SCFA Analysis Using HPLC-HESI-MS/MS

To support the findings of our gas-phase VFA analysis results, The C2–C7 VFAs from liquid samples of the collected microbial cultures were also evaluated with HPLC-HESI-MS/MS analysis via a derivatization method. Our results showed that compared to the control group, slightly increased levels of C_4_ VFAs from the test group of gut microbial culture can be observed (Figure S1C,D). However, a statistical significance was not reached, which may be due to the sample loss during the sample derivatization process or the semi-quantitative nature of both types of analyses. This may indicate that headspace analysis using SESI-HRMS could be used for the accurate evaluation of VFA-related health outcomes based on its high sensitivity. Furthermore, the exported peak areas of VFAs (C_2_–C_7_) were normalized using OD values and the Spearman’s correlation coefficients between SESI-HRMS and HPLC-HESI-MS/MS analysis was performed. The results were presented in Figure S2. All studied VFAs (C_2_–C_7_) strongly, positively correlated with each other between two analytical techniques (*p* < 0.001). It also worth noting that while isomers can be separated on the stationary phase during the HPLC-HESI-MS/MS analysis, such as the separation and detection isobutyric acid and butyric acid (C_4_-VFAs), isovaleric acid, and valeric acid (C_5_-VFAs), and isocaproic acid and hexanoic acid (C_6_-VFAs), the SESI-HRMS analysis of isomers are still relatively challenging, and therefore, most of the current studies still prefer to combine the isomer signals as we reported in this study.

Despite the fact that such limitation of unseparated isomers has been observed in the use of SESI-MS, cumulative numbers of studies across various fields demonstrated the promising bioanalytical potentials of SESI-MS for studying headspace volatilome present in relatively low concentrations, in comparison to standard analytical tools [2,5,6,7]. Typically, a headspace solid-phase microextraction coupled to gas chromatography-mass spectrometry (HS-SPME/GC-MS) technique requires up to one hour for extraction and analysis [50]. Consequently, the extended steps can be attributed to heavily contaminated profiles by environmental VOCs [51]. In this context, SESI-MS can be an effective tool for sampling microbial VOCs based on its advantageous features, including spontaneous response and high sensitivity in a time- and cost-efficient manner. In addition, the connection of an ion mobility spectrometry (IMS), which enables to separate VOCs according to size and structure of sample analytes, to SESI-MS technique can be considered to improve identification and quantitation of bacterial VOCs [52].

## 3. Materials and Methods

### 3.1. Chemicals

Chemical, including LC/MS grade methanol and acetonitrile, analytical grade formic acid, Gifu Anaerobic Broth (GAM broth), and ampicillin sodium salt, 3-Nitrophenylhydrazine hydrochloride (3NPH·HCl), N-(3-dimethylaminopropyl)-N’-ethylcarbodiimide (EDC·HCl), and pyridine were purchased from Fisher Scientific (Pittsburgh, PA, USA). The volatile free fatty acid (VFA) standard mix (certified reference material CRM46975) was purchased from Supelco (Bellefonte, PA, USA).

### 3.2. Setup and Optimization of Secondary Electrospray Ionization (SESI)

The volatile free fatty acid (VFA) standard mixture, consisting of acetic acid (C_2_), propionic acid (C_3_), isobutyric acid and butyric acid (C_4_), isovaleric acid and valeric acid (C_5_), isocaproic acid and hexanoic acid (C_6_), and heptanoic acid (C_7_), was prepared in deionized water (50 µm) for optimizing the condition of headspace analysis using a novel prototype of secondary electrospray ionization (SESI), named SuperSESI (Fossil Ion Tech, Madrid, Spain). The Super SESI was used in this study and directly connected to a Thermo Q-Exactive high-resolution mass spectrum (HRMS) (Thermo Scientific, San Jose, CA, USA). The Super SESI source was optimized for the headspace volatile analysis of gut microbial cultures. The initial SESI parameters are as following: SESI pressure of 1 bar, and temperatures of sample inlet and ion chamber were set at 70 °C and 80 °C, respectively.

Selection of spray solvent. To compare the efficiency of real-time headspace analysis using SESI-HRMS, four types of nano-electrospray solvents, including deionized (DI) water, 60%, 40%, and 20% methanol, which were prepared in DI water with 0.1% formic acid, were prepared to generate the seeding ESI that can later transfer the charges to the VFAs present in the headspace of the standard mixture. After the selection of solvent types, the additive which contributes to the ionization efficiency was also assessed by testing formic acid at different concentrations of 0.01, 0.05, and 0.1% (*v*/*v*).

Temperatures of sample inlet. To determine the optimal temperature of the sample inlet which was made of a Teflon tube (length 80 cm, i.d. 1.48 mm), various temperatures such as 70, 90, 110, 130, and 150 °C were examined along with the optimized electrospray solvent.

Temperatures of the ion chamber. To evaluate the temperature of the ion chamber for the maximum signal intensity, 75, 85, and 95 °C were screened using the above-optimized conditions.

Comparison of bar pressure. Finally, the effect of SESI pressure (bar) was tested by comparing the results based on tested values of 0.5, 0.7, 1, 1.3, and 1.5 bar with the other optimal SESI conditions.

### 3.3. High-Resolution Mass Spectrometry (HRMS)

The SuperSESI source was coupled to the Q Exactive MS system (Thermo Fisher, Waltham, MA, USA) with the following instrumental parameters: sheath gas flow rate, 60; auxiliary gas flow rate, 2; spray voltage, 3.5 kV; capillary temperature, 275 °C; and S-lens RF level, 60.0. The MS was controlled directly using Q Exactive Tune software (version 2.9) in full MS mode. The scan range of negative ionization modes was set from 50 to 500 m/z; microscans of 1; AGC target of 1e6; and maximum injection time of 100 ms) with a high resolution of 140,000.

### 3.4. Growth Condition of Microbial Cultures and Antibiotics Treatment

Fecal samples with gut microbes for the described experiments were donated by two healthy volunteers that were not subjected to antibiotic treatment in the past six months. Approval was obtained from the Institute Review Board (IRB) committee before the study, and informed consent were obtained from the healthy fecal donors. The pooled fresh human fecal samples were immediately used to culture gut microbiota following a generic extraction procedure. The fecal content was suspended in pre-reduced PBS with 0.1% cysteine by vortexing. The diluted suspension was plated on Gifu Anaerobic (GAM) Agar plate (HiMedia Laboratories LLC, West Chester, PA, USA) for 48 h at 37 °C in an anaerobic environment inside a type A vinyl anaerobic chamber (COY lab, Grass Lake, Michigan). The anaerobic chamber was operated according to the manufacturer recommended protocols. Colonies from the agar plate were selected by using a sterile wire loop and incubated in GAM broth for 48 h under the same condition. To examine the impact of antibiotic treatment on headspace metabolic profiles of the gut microbiome, frozen stock of pooled gut microbes from human fecal samples were used in this study and were first cultured in the Gifu anaerobic (GAM) broth culture for 24 h at 37 °C under strict anaerobic condition using a Coy Anaerobic Chamber with incubator (Coy Laboratory Products INC, Grass Lake, MI, USA). Then, the ampicillin stock solution was prepared in autoclaved DI water, and 100 µL was added into the 24-h cultures to reach a final concentration of 1 mg/L. For the control group, 100 µL of water was added into the 24-h cultures. Then, test and control cultures were incubated for six additional hours before the VOC measurement was performed [4]. The optical density (OD) values before and after the treatment of ampicillin were measured using spectrophotometers at 595 nm.

### 3.5. Real-Time Analysis of Headspace Volatile in Gut Microbial Cultures

The headspace of gut microbial cultures was analyzed in real-time by SESI-HRMS [4]. Briefly, 10 mL of microbial culture was placed in the 100 mL glass bottle and sealed with a three-port GL45 cap. Fully sealed glass bottle with a three-port GL45 cap was immediately transferred for headspace of SESI-HRMS analysis. To introduce the headspace volatiles of sample bottles to the SESI-HRMS, clean nitrogen was used as a carrier gas. The fraction of gas flowing into the SESI ionizer was fixed at 0.3 L/min by setting an excess of 0.4 L/min over the flow ingested by the mass spectrometer and the exhaust mass flow controller at 0.7 L/min. Three biological replicates were analyzed, and each replicate consists of three technical replicates by scanning the headspace samples for 15 s each.

### 3.6. HPLC-HESI-MS/MS Analysis of Microbial Cultures

#### 3.6.1. Extraction of C2–C7 Volatile Fatty Acids (VFAs) of Microbial Cultures

Immediately after the SESI-HRMS analysis of ampicillin treatment, 1 mL of microbial cultures was placed into 2 mL tubes and kept at −80 °C until further metabolite extraction. The previously published method was modified for the analysis of C2–C7 volatile fatty acids from the liquid cultures [53]. Briefly, the collected supernatant was centrifuged at 14,000 rpm for 10 min. Then 40 µL of aliquot was mixed with 200 mM 3-nitrophenyl-hydrazine (3NPH) and 120 mM N-(3-dimethylaminopropyl)-N’-ethylcarbodiimide (EDC·HCl) was prepared in 6% pyridine solution, and the mixture was vortexed and incubated for 30 min at 40 °C to generate 3NPH-FAA derivatives. Finally, the mixture was filled up to 2 mL with 10% aqueous acetonitrile after cooling on ice for 1 min and aliquoted into LC vials for HPLC-HESI-MS/MS analysis.

#### 3.6.2. HPLC-HESI-MS/MS Analysis

Sample analyses were performed on a high-performance liquid chromatography system coupled with heat electrospray ionization tandem mass spectrometer (HPLC-HESI-MS/MS) in negative mode. A 10-µL aliquot of the solution is injected and separated on an ACQUITY UPLC CSH C18 column (2.1 mm × 100 mm, 1.7 μm; Waters Corporation) using binary mobile phase of 0.01% aqueous formic acid (A) and 0.01% formic acid in acetonitrile (B). The separation was achieved with the following gradient program: 0 min, 15% B; 2.1 min, 15% B; 11 min, 55% B; 11.1 min 100% B; 12 min, 15% B; 15 min, 15% and the flow rate at 0.35 mL/min. Running time was 15 min, and the column temperature was kept constant at 40 °C.

### 3.7. Data Processing and Statistical Analysis

Xcalibur version 4.0 (Thermo Fisher Scientific, Waltham, MA, USA) was used to process both SESI-MS and HPLC-HESI-MS/MS data set. The signal intensities of spectral peaks were manually examined using the Quan browser module and exported to Excel spreadsheets. The peak areas were normalized by corresponding OD values of the microbes. Significant differences between control and test groups were assessed with a Student’s t-test (*p* < 0.05). The multiple mean comparisons (*p* < 0.05) were carried out by one-way analysis of variance (ANOVA) with Tukey’s Honest Significant Difference (HSD) test using SPSS software (v. 26, BM SPSS Statistics, IBM Corp., Chicago, IL, USA). Annotation of six m/z features (C_2_‒C_7_) was carried according to putative identification of fingerprint peaks observed in the full scans using real-time SESI-HRMS by comparing m/z features to authentic standards and existing literature [4]. For HPLC-HESI-MS/MS, nine m/z values, including separated isomers (C_4_, C_5_, and C_6_) on a stationary phase, were annotated based on authentic standards and previous literature [54].

## 4. Conclusions

In summary, herein, we optimized the real-time SESI-HRMS method for the analysis of C_2_-C_7_-VFAs from the headspace of gut microbial cultures after six hours post-treatment of ampicillin (1 mg/L). To the best of our knowledge, this is the first comprehensive study to showcase the impact of SESI parameters, including the selection of an electrospray solvent with a specific concentration of an additive, temperatures of sample inlet and ion chamber, and SESI spray pressure, on the signal intensities of SESI-HRMS analysis. Notably, our findings indicate that the optimization of SESI parameters may be pre-required to obtain the maximum and accurate intensity based on our observation that as the five-times higher signal intensity can be obtained after systematic optimization in comparison to the results from the least optimized condition. Moreover, the excellent linearity of calibration curves using a C_2_–C_7_-VFAs standard solution implies its robustness for headspace VOCs analysis. Furthermore, as an application example, considerably elevated levels of C_4_ and C_7_ VFAs were observed in response to antibiotics treatment compared with control during the SESI-HRMS analysis of headspace of gut microbial cultures. However, the effect of mass spectrometry conditions on the signal intensity of VFAs may need to be further investigated, since we only focused on optimizing SESI parameters in this study. In addition, adding internal standards for headspace VOCs analysis in gut microbial cultures may need to be considered to improve quantitation accuracy and precision. Overall, our findings suggest that the SESI-HRMS technique, after appropriate optimization, is a promising and suitable bioanalytical tool for the gut microbial VOCs analysis in a robust, non-invasive, and sensitive manner.

## Figures and Tables

**Table 1:** Mass accuracy for C_2_–C_7_ volatile fatty acids (VFAs) ions detected in the headspace of gut microbial cultures using SESI-HRMS in negative mode with a resolution of 140,000.

Compounds ^a^	Formula	Theoretical m/z [M-H]^−^	Measured m/z [M-H]^−^	Mass Error (ppm)
Acetic acid (C_2_-VFA)	C_2_H_4_O_2_	59.0139	59.0138	−0.9
Propionic acid (C_3_-VFA)	C_3_H_6_O_2_	73.0295	73.0296	1.3
Butyric acid/Isobutyric acid (C_4_-VFA)	C_4_H_8_O_2_	87.0452	87.0453	1.7
Valeric acid/Isovaleric acid (C_5_-VFA)	C_5_H_10_O_2_	101.0608	101.0609	1.0
Hexanoic acid/Isocarporic acid (C_6_-VFA)	C_6_H_12_O_2_	115.0765	115.0765	0.4
Heptanoic acid (C_7_-VFA)	C_7_H_14_O_2_	129.0921	129.0922	0.8

^a^ Identification was confirmed by HPLC-HESI-MS/MS spectrum using the authentic standard.

## Supplementary Materials

The following are available online at https://www.mdpi.com/2218-1989/10/9/351/s1, Figure S1: The effect of the ampicillin with a moderate level (10 mg/L) on abundance of C_2_–C_7_ volatile fatty acids (VFAs) in headspace of collected gut microbial cultures was estimated using a HPLC-HESI-MS/MS. There was no significant difference between control and test groups. Three biological replicates were used to examine the effect of optimized and least optimized SESI conditions on signal intensities of volatile fatty acids (C_2_‒C_7_) in the headspace of gut microbial cultures by performing the Student t-test. Figure S2: The exported peak areas of VFAs (C_2_–C_7_) were normalized using OD values and the correlation analysis between SESI-HRMS and HPLC-HESI-MS/MS analysis was performed. Spearman’s correlation coefficients between the results obtained by SESI-HRMS and HPLC-HESI-MS/MS were reported here. All studied VFAs (C_2_–C_7_) strongly positively correlated with each other between two analytical techniques (*p* < 0.001).

## References

[1] Crespo E., Cristescu S.M., de Ronde H., Kuijper S., Kolk A.H., Anthony R.M., Harren F.J. (2011). Proton Transfer Reaction Mass Spectrometry detects rapid changes in volatile metabolite emission by Mycobacterium smegmatis after the addition of specific antimicrobial agents. J. Microbiol. Methods.

[2] O’Hara M., Mayhew C. (2009). A preliminary comparison of volatile organic compounds in the headspace of cultures of *Staphylococcus aureus* grown in nutrient, dextrose and brain heart bovine broths measured using a proton transfer reaction mass spectrometer. J. Breath Res..

[3] Slade E.A., Thorn R., Lovering A.M., Young A., Reynolds D.M. (2017). In vitro discrimination of wound-associated bacteria by volatile compound profiling using selected ion flow tube-mass spectrometry. J. Appl. Microbiol..

[4] Lee J.H.J., Jayaprakasha G.K., Rush C.M., Crosby K.M., Patil B.S. (2018). Production system influences volatile biomarkers in tomato. Metabolomics.

[5] Li H., Zhu J. (2018). Differentiating antibiotic-resistant staphylococcus aureus using secondary electrospray ionization tandem mass spectrometry. Anal. Chem..

[6] Rioseras A.T., Gomez D.G., Ebert B.E., Blank L.M., Ibáñez A.J., Sinues P.M. (2017). Comprehensive real-time analysis of the yeast Volatilome. Sci. Rep..

[7] Farrell R.R., Fahrentrapp J., García-Gómez D., Sinues P.M.-L., Zenobi R. (2017). Rapid fingerprinting of grape volatile composition using secondary electrospray ionization orbitrap mass spectrometry: A preliminary study of grape ripening. Food Control.

[8] Weber R., Haas N., Baghdasaryan A., Bruderer T., Inci D., Micic S., Perkins N., Spinas R., Zenobi R., Moeller A. (2020). Volatile organic compound breath signatures of children with cystic fibrosis by real-time SESI-HRMS. ERJ Open Res.

[9] Martínez-Lozano P., Rus J., de la Mora G.F., Hernández M., de la Mora J.F. (2009). Secondary electrospray ionization (SESI) of ambient vapors for explosive detection at concentrations below parts per trillion. J. Am. Soc. Mass Spectr..

[10] Gaugg M.T., Gomez D.G., Barrios-Collado C., Vidal-de-Miguel G., Kohler M., Zenobi R., Martinez-Lozano Sinues P. (2016). Expanding metabolite coverage of real-time breath analysis by coupling a universal secondary electrospray ionization source and high resolution mass spectrometry—A pilot study on tobacco smokers. J. Breath Res..

[11] Fenn J.B., Mann M., Meng C.K., Wong S.F., Whitehouse C.M. (1989). Electrospray ionization for mass spectrometry of large biomolecules. Science.

[12] Chen Y.H., Hill Jr H.H., Wittmer D.P. (1994). Analytical merit of electrospray ion mobility spectrometry as a chromatographic detector. J. Microcolumn Sep..

[13] Vidal-de-Miguel G., Macia M., Pinacho P., Blanco J. (2012). Low-sample flow secondary electrospray ionization: Improving vapor ionization efficiency. Anal. Chem..

[14] Barrios-Collado C., Vidal-de-Miguel G., Sinues P.M.-L. (2016). Numerical modeling and experimental validation of a universal secondary electrospray ionization source for mass spectrometric gas analysis in real-time. Sens. Actuators B Chem..

[15] Zhang Q., Lin L., Yu Q., Wang X. (2020). Exploiting the native inspiratory ability of a mass spectrometer to improve analysis efficiency. RSC Adv..

[16] Lavelle A., Sokol H. (2020). Gut microbiota-derived metabolites as key actors in inflammatory bowel disease. Nat. Rev. Gastroenterol. Hepatol..

[17] Ho K.M., Kalgudi S., Corbett J.M., Litton E. (2020). Gut microbiota in surgical and critically ill patients. Anaesth. Intensive Care.

[18] Holota Y., Dovbynchuk T., Kaji I., Vareniuk I., Dzyubenko N., Chervinska T., Zakordonets L., Stetska V., Ostapchenko L., Serhiychuk T. (2019). The long-term consequences of antibiotic therapy: Role of colonic short-chain fatty acids (SCFA) system and intestinal barrier integrity. PLoS ONE.

[19] Raposo F., Borja R., Cacho J., Mumme J., Orupõld K., Esteves S., Noguerol-Arias J., Picard S., Nielfa A., Scherer P. (2013). First international comparative study of volatile fatty acids in aqueous samples by chromatographic techniques: Evaluating sources of error. Trends Anal. Chem..

[20] Lamichhane S., Sen P., Dickens A.M., Orešič M., Bertram H.C. (2018). Gut metabolome meets microbiome: A methodological perspective to understand the relationship between host and microbe. Methods.

[21] Harsch I.A., Konturek P.C. (2018). The role of gut microbiota in obesity and type 2 and type 1 diabetes mellitus: New insights into “old” diseases. Med. Sci..

[22] Velikonja A., Lipoglavšek L., Zorec M., Avguštin G. (2019). Alterations in gut microbiota composition and metabolic parameters after dietary intervention with barley beta glucans in patients with high risk for metabolic syndrome development. Anaerobe.

[23] Vetrani C., Costabile G., Luongo D., Naviglio D., Rivellese A.A., Riccardi G., Giacco R. (2016). Effects of whole-grain cereal foods on plasma short chain fatty acid concentrations in individuals with the metabolic syndrome. Nutrition.

[24] Hald S., Schioldan A.G., Moore M.E., Dige A., Lærke H.N., Agnholt J., Bach Knudsen K.E., Hermansen K., Marco M.L., Gregersen S. (2016). Effects of arabinoxylan and resistant starch on intestinal microbiota and short-chain fatty acids in subjects with metabolic syndrome: A randomised crossover study. PLoS ONE.

[25] Kasote D.M., Jayaprakasha G., Patil B.S. (2018). Encapsulation of Polyphenols: An Effective Way to Enhance Their Bioavailability for Gut Health. Advances in Plant Phenolics: From Chemistry to Human Health.

[26] Manuzak J.A., Zevin A.S., Cheu R., Richardson B., Modesitt J., Hensley-McBain T., Miller C., Gustin A.T., Coronado E., Gott T. (2020). Antibiotic-induced microbiome perturbations are associated with significant alterations to colonic mucosal immunity in *rhesus macaques*. Mucosal Immunol..

[27] Graversen K.B., Bahl M.I., Larsen J.M., Ballegaard A.R., Licht T.R., Bogh K.L. (2020). Short-Term Amoxicillin-Induced Perturbation of the Gut Microbiota Promotes Acute Intestinal Immune Regulation in Brown Norway Rats. Front. Microbiol..

[28] Bruderer T., Gaisl T., Gaugg M.T., Nowak N., Streckenbach B., Müller S., Moeller A., Kohler M., Zenobi R. (2019). On-line analysis of exhaled breath: Focus review. Chem. Rev..

[29] Kiontke A., Oliveira-Birkmeier A., Opitz A., Birkemeyer C. (2016). Electrospray ionization efficiency is dependent on different molecular descriptors with respect to solvent pH and instrumental configuration. PLoS ONE.

[30] Tejero Rioseras A., Singh K.D., Nowak N., Gaugg M.T., Bruderer T., Zenobi R., Sinues P.M. (2018). Real-Time Monitoring of Tricarboxylic Acid Metabolites in Exhaled Breath. Anal. Chem..

[31] Meier L., Berchtold C., Schmid S., Zenobi R. (2012). Sensitive detection of drug vapors using an ion funnel interface for secondary electrospray ionization mass spectrometry. J. Mass Spectrom..

[32] Meier L., Berchtold C., Schmid S., Zenobi R. (2012). High mass resolution breath analysis using secondary electrospray ionization mass spectrometry assisted by an ion funnel. J. Mass Spectrom..

[33] Sinues P.M.-L., Criado E., Vidal G. (2012). Mechanistic study on the ionization of trace gases by an electrospray plume. Int. J. Mass Spectrom..

[34] Singh K.D., Tancev G., Decrue F., Usemann J., Appenzeller R., Barreiro P., Jauma G., Macia Santiago M., Vidal de Miguel G., Frey U. (2019). Standardization procedures for real-time breath analysis by secondary electrospray ionization high-resolution mass spectrometry. Anal. Bioanal. Chem..

[35] Rioseras A.T., Gaugg M.T., Sinues P.M.-L. (2017). Secondary electrospray ionization proceeds via gas-phase chemical ionization. Anal. Methods.

[36] Sinues P.M., Kohler M., Zenobi R. (2013). Monitoring diurnal changes in exhaled human breath. Anal. Chem..

[37] Bean H.D., Mellors T.R., Zhu J., Hill J.E. (2015). Profiling aged artisanal Cheddar cheese using secondary electrospray ionization mass spectrometry. J. Agric. Food Chem..

[38] Wolf J.-C., Schaer M., Siegenthaler P., Zenobi R. (2015). Direct quantification of chemical warfare agents and related compounds at low ppt levels: Comparing active capillary dielectric barrier discharge plasma ionization and secondary electrospray ionization mass spectrometry. Anal. Chem..

[39] Cristoni S., Rossi Bernardi L., Guidugli F., Tubaro M., Traldi P. (2005). The role of different phenomena in surface-activated chemical ionization (SACI) performance. J. Mass Spectrom..

[40] Zhao J., Nian L., Kwok L., Sun T. (2017). Reduction in fecal microbiota diversity and short-chain fatty acid producers in methicillin-resistant Staphylococcus aureus infected individuals as revealed by PacBio single molecule, real-time sequencing technology. Eur. J. Clin. Microbiol. Infectious Dis..

[41] Canani R.B., Di Costanzo M., Leone L., Pedata M., Meli R., Calignano A. (2011). Potential beneficial effects of butyrate in intestinal and extraintestinal diseases. World J. Gastroenterol. WJG.

[42] Jung T.-H., Park J.H., Jeon W.-M., Han K.-S. (2015). Butyrate modulates bacterial adherence on LS174T human colorectal cells by stimulating mucin secretion and MAPK signaling pathway. Nutr. Res. Pract..

[43] Peng L., Li Z.-R., Green R.S., Holzman I.R., Lin J. (2009). Butyrate enhances the intestinal barrier by facilitating tight junction assembly via activation of AMP-activated protein kinase in Caco-2 cell monolayers. J. Nutr..

[44] Weir T.L., Manter D.K., Sheflin A.M., Barnett B.A., Heuberger A.L., Ryan E.P. (2013). Stool microbiome and metabolome differences between colorectal cancer patients and healthy adults. PLoS ONE.

[45] Zhang Y.-J., Li S., Gan R.-Y., Zhou T., Xu D.-P., Li H.-B. (2015). Impacts of gut bacteria on human health and diseases. Int. J. Mol. Sci..

[46] Høverstad T., Carlstedt-Duke B., Lingaas E., Midtvedt T., Norin K., Saxerholt H., Steinbakk M. (1986). Influence of ampicillin, clindamycin, and metronidazole on faecal excretion of short-chain fatty acids in healthy subjects. Scand. J. Gastroenterol..

[47] Willing B.P., Russell S.L., Finlay B.B. (2011). Shifting the balance: Antibiotic effects on host–microbiota mutualism. Nat. Rev. Microbiology.

[48] Zhao X., Jiang Z., Yang F., Wang Y., Gao X., Wang Y., Chai X., Pan G., Zhu Y. (2016). Sensitive and simplified detection of antibiotic influence on the dynamic and versatile changes of fecal short-chain fatty acids. PLoS ONE.

[49] El-Ansary A., Bhat R.S., Al-Daihan S., Al Dbass A.M. (2015). The neurotoxic effects of ampicillin-associated gut bacterial imbalances compared to those of orally administered propionic acid in the etiology of persistent autistic features in rat pups: Effects of various dietary regimens. Gut Pathog..

[50] Tait E., Perry J.D., Stanforth S.P., Dean J.R. (2014). Identification of volatile organic compounds produced by bacteria using HS-SPME-GC–MS. J. Chromatogr. Sci..

[51] Timm C.M., Lloyd E.P., Egan A., Mariner R., Karig D. (2018). Direct Growth of Bacteria in Headspace Vials Allows for Screening of Volatiles by Gas Chromatography Mass Spectrometry. Front. Microbiol..

[52] Lapthorn C., Pullen F., Chowdhry B.Z. (2013). Ion mobility spectrometry-mass spectrometry (IMS-MS) of small molecules: Separating and assigning structures to ions. Mass Spectrom. Rev..

[53] Hodges J.K., Zhu J., Yu Z., Vodovotz Y., Brock G., Sasaki G.Y., Dey P., Bruno R.S. (2020). Intestinal-level anti-inflammatory bioactivities of catechin-rich green tea: Rationale, design, and methods of a double-blind, randomized, placebo-controlled crossover trial in metabolic syndrome and healthy adults. Contemp. Clin. Trials Commun..

[54] Han J., Lin K., Sequeira C., Borchers C.H. (2015). An isotope-labeled chemical derivatization method for the quantitation of short-chain fatty acids in human feces by liquid chromatography-tandem mass spectrometry. Anal. Chim. Acta.

