# Supplementary material for: Optimizing Secondary Electrospray Ionization High-Resolution Mass Spectrometry (SESI-HRMS) for the Analysis of Volatile Fatty Acids from Gut Microbiome
Source: Metabolites. 2020 Aug 28;10(9):351. doi: 10.3390/metabo10090351 (PMC7570293; doi:10.3390/metabo10090351)
Supplement: Supplementary file 1 [file metabolites-10-00351-s001.pdf]

Supplementary information

Optimizing secondary electrospray ionization high-resolution mass spectrometry (SESI-HRMS) for the analysis of volatile fatty acids from gut microbiome

Jisun H.J. Lee[1,2] and Jiangjiang Zhu[1,2*]

[1] Department of Human Sciences, The Ohio State University, Campbell Hall, 1787 Neil Avenue, Columbus, OH 43210, USA; lee.9639@osu.edu

[2] James Comprehensive Cancer Center, The Ohio State University, Wiseman Hall, 400 W 12th Ave, Columbus, OH 43210, USA

* Correspondence: zhu.2484@osu.edu

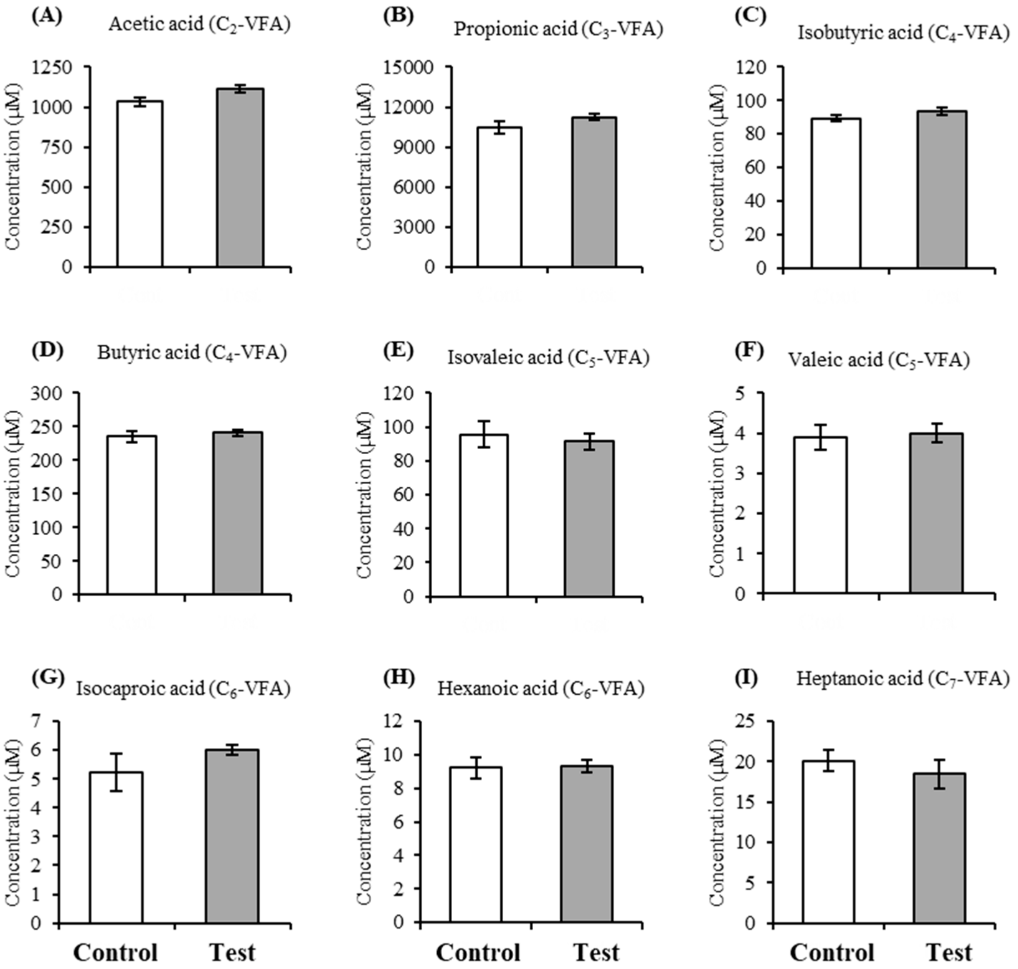

Figure S1. The effect of the ampicillin with a moderate level (10 mg/L) on abundance of C_2-C_7 volatile fatty acids (VFAs) in headspace of collected gut microbial cultures was estimated using a HPLC-HESI-MS/MS. There was no significant difference between control and test groups. Three biological replicates were used to examine the effect of optimized and least optimized SESI conditions on signal intensities of volatile fatty acids (C_2–C_7) in the headspace of gut microbial cultures by performing the Student t-test.

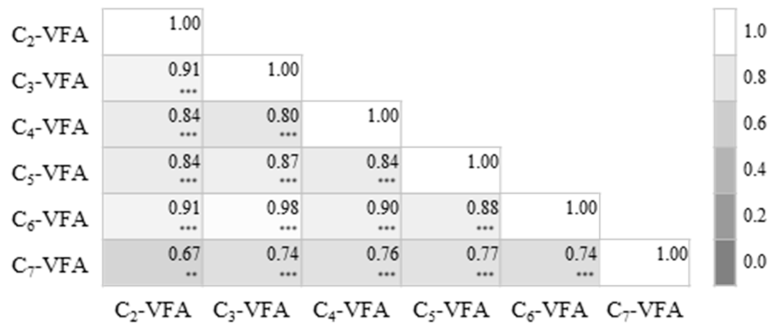

Figure S2. The exported peak area of VFAs (C_2-C_7) were normalized using OD values and the correlation analysis between SESI-HRMS and HPLC-HESI-MS/MS analysis was performed. Spearman's correlation coefficients between the results obtained by SESI-HRMS and HPLC-HESI-MS/MS were reported here. All studied VFAs (C_2-C_7) strongly positively correlated with each other between two analytical techniques ($p < 0.001$).
